# Ultrafiltration and Nanofiltration for the Removal of Pharmaceutically Active Compounds from Water: The Effect of Operating Pressure on Electrostatic Solute—Membrane Interactions

**DOI:** 10.3390/membranes13080743

**Published:** 2023-08-19

**Authors:** Alexandre Giacobbo, Isabella Franco Pasqualotto, Rafael Cabeleira de Coronel Machado Filho, Miguel Minhalma, Andréa Moura Bernardes, Maria Norberta de Pinho

**Affiliations:** 1 Post-Graduation Program in Mining, Metallurgical and Materials Engineering (PPGE3M), Federal University of Rio Grande do Sul (UFRGS), Av. Bento Gonçalves n. 9500, Porto Alegre 91509-900, Brazil; isabellafp@outlook.com.br (I.F.P.); cabeleirafilho@gmail.com (R.C.d.C.M.F.); amb@ufrgs.br (A.M.B.); 2 Centre of Physics and Engineering of Advanced Materials (CeFEMA), Instituto Superior Técnico, University of Lisbon, Av. Rovisco Pais, n. 1, 1049-001 Lisbon, Portugal; mminhalma@deq.isel.ipl.pt; 3 Chemical Engineering Department, Instituto Superior de Engenharia de Lisboa, Instituto Politécnico de Lisboa, Rua Conselheiro Emídio Navarro, 1, 1959-007 Lisboa, Portugal; 4 Chemical Engineering Department, Instituto Superior Técnico, University of Lisbon, Av. Rovisco Pais, n. 1, 1049-001 Lisbon, Portugal

**Keywords:** ultrafiltration, nanofiltration, electrostatic interaction, contaminants of emerging concern, phac, antibiotic, water, wastewater, membrane separation process

## Abstract

The present work investigates nanofiltration (NF) and ultrafiltration (UF) for the removal of three widely used pharmaceutically active compounds (PhACs), namely atenolol, sulfamethoxazole, and rosuvastatin. Four membranes, two polyamide NF membranes (NF90 and NF270) and two polyethersulfone UF membranes (XT and ST), were evaluated in terms of productivity (permeate flux) and selectivity (rejection of PhACs) at pressures from 2 to 8 bar. Although the UF membranes have a much higher molecular weight cut-off (1000 and 10,000 Da), when compared to the molecular weight of the PhACs (253–482 Da), moderate rejections were observed. For UF, rejections were dependent on the molecular weight and charge of the PhACs, membrane molecular weight cut-off (MWCO), and operating pressure, demonstrating that electrostatic interactions play an important role in the removal of PhACs, especially at low operating pressures. On the other hand, both NF membranes displayed high rejections for all PhACs studied (75–98%). Hence, considering the optimal operating conditions, the NF270 membrane (MWCO = 400 Da) presented the best performance, achieving permeate fluxes of about 100 kg h^−1^ m^−2^ and rejections above 80% at a pressure of 8 bar, that is, a productivity of about twice that of the NF90 membrane (MWCO = 200 Da). Therefore, NF270 was the most suitable membrane for this application, although the tight UF membranes under low operating pressures displayed satisfactory results.

## 1. Introduction

Contaminants of emerging concern (CEC) are chemical or biological substances with potential risks to humans and the environment [1,2]. These contaminants encompass personal care products, pharmaceutically active compounds (PhACs), surfactants, flame retardants, and antibiotic-resistant genes, as well as other non-regulated substances [3,4]. They have been introduced into the environment through the disposal of wastewater [5,6] since conventional treatment processes commonly used in wastewater treatment plants (WWTPs) are not effective in removing these contaminants [7,8]. 

Based on these pieces of evidence, advanced technologies like adsorption [9,10], advanced oxidative processes (AOPs) [11,12], and membrane technologies [13,14] have been investigated for removing PhACs from water and wastewater. However, thanks to characteristics such as low energy consumption, ease of scaling up, no additives requirements, mild operating conditions, and separation efficiency, membrane technologies have gained prominence over the others [15,16].

Among the membrane technologies, RO and NF have been most used in the treatment of water and wastewater containing PhACs because most of these contaminants have a molecular weight (MW) in the range of the molecular weight cut-off (MWCO) of RO and NF membranes [17,18], but there are also some studies employing ultrafiltration (UF) for these purposes [19,20]. 

In fact, studies have shown that these technologies increase the safety of the treatment system and reduce the risks of environmental and human contamination [21,22]. Because it uses the tightest membranes, RO provides a higher level of safety to the treatment but also presents greater implantation and operation costs due to the lowest permeate fluxes and highest energy consumption compared to the other membrane technologies [23]. So, NF is currently the membrane technology showing the best performance in the treatment of water and wastewater containing PhACs, as it has a permeate flux greater than RO and high removals for these contaminants, generally greater than 80%, even using membranes with MWCO greater than the PhACs’ MW [24,25]. 

In a study assessing a loose NF membrane (TFC-SR2, from Koch Membrane Systems, Wilmington, MA, USA) for removing three PhACs, sulfamethoxazole (SMX), carbamazepine, and ibuprofen, from water, Nghiem et al. [26] achieved removal rates considerably high (50–85%) for all PhACs tested. The authors concluded that the rejection of the ionizable PhACs, sulfamethoxazole and ibuprofen, was strongly influenced by the solution pH and ionic strength, while the rejection of the non-ionizable PhAC, carbamazepine, was independent of the solution chemistry. That is, the rejection of PhACs increases dramatically as the compound changes from a neutral to a negatively charged form when the pH of the solution rises above its pKa value. 

Licona et al. [25] evaluated the potential of a tight NF membrane (NF90 from Dow FilmTec, Edina, MN, USA) in removing acetaminophen, caffeine, diclofenac, dipyrone, and ibuprofen from water, reaching removal rates greater than 88% for all PhACs evaluated under all operating conditions tested (pH: 4–7; pressure: 5–20 bar). The authors pointed out that the steric hindrance mechanism governs the removal of non-ionizable PhACs (acetaminophen and caffeine), while both the steric hindrance mechanism and electrostatic interactions influence the removal of ionizable PhACs (diclofenac, dipyrone, and ibuprofen).

Taheri et al. [27] studied the performance of a loose NF membrane (NF33, from ASPRINN, Whippany, NJ, USA) in retaining the PhAC atenolol (ATN) from simple and binary aqueous solutions under different conditions (pH: 3–9; pressure: 3–11; feed flux: 8.15–48.75 L m^−2^ h^−1^; ATN concentration: 10–60 mg L^−1^; and co-existing salts: NaCl, Na_2_SO_4_ and CaCl_2_). The authors reported an increment in ATN removal with increasing solution pH, reaching the highest removal at pH 9 (70.9%), which slightly decreased with increasing feed ATN concentration, but increased again with rising feed flux and applied pressure. 

Heo et al. [19] investigated three UF membranes (UF5K, UF10K, and UF30K, from Koch Membrane Systems Inc., Wilmington, MA, USA), having 5000, 10,000, and 30,000 Da MWCO, respectively, for removing an endocrine disruptor (bisphenol A) and a PhAC (17 β-estradiol) from aqueous solutions containing natural organic matter (NOM), single-walled carbon nanotubes (SWNTs), or NOM + SWNT. The authors reported a linear fitting between the retention and adsorption of bisphenol A and 17 β-estradiol, which indicates that retention by the UF membranes was mainly due to the adsorption of bisphenol A and 17 β-estradiol in NOM, SWNTs, and/or onto the membrane.

In another study, Shakak et al. [20] evaluated nanocomposite UF membranes (polysulfone/polyvinylpyrrolidone/silica nanoparticles), prepared by themselves in the laboratory using the phase inversion technique, for the removal of amoxicillin from aqueous solutions. The authors reported an improvement in amoxicillin rejection, from 66.52% to 89.81%, by increasing silica nanoparticles from 0 to 4 wt%.

Indeed, studies have shown that a variety of factors (membrane properties, solution chemistry, and operating conditions) can play an important role in membrane technologies for the removal of PhACs from water and wastewater. In tighter membranes, such as RO, membrane/solute interactions have been pointed out as the main factor for the removal of PhACs, while for membranes with larger pores, such as UF, the chemistry of the solution, more precisely through the adsorption of PhACs in NOM, plays the most important role in the removal of PhACs. In the range between RO and UF, and namely in NF, all factors act together to retain theses contaminants. So, although the role of electrostatic interactions in the removal of PhACs by NF membranes is already a consensus, additional studies on the removal of these contaminants by UF and NF membranes are still necessary since, in particular, the selectivity of the UF membranes for PhACs is often attributed to adsorption processes, either in the NOM [19,28] present in the water or the adsorbents [29,30] and/or coagulants/flocculants [31] added along the treatment process. Therefore, this study aims to provide further insights into the mechanisms involved in the removal of PhACs by UF and NF. For this purpose, two tight UF membranes (1000 and 10,000 Da) and two NF membranes (200 and 400 Da) were investigated in terms of productivity (permeate flux) and selectivity for PhACs under different operating conditions and using three PhACs with different physicochemical characteristics. Concentration polarization was assessed by the film theory and the transport of PhACs across UF and NF membranes was investigated by solution-diffusion (SD) and Spiegler–Kedem–Katchalsky (SKK) models. 

## 2. Materials and Methods

### 2.1. Membranes

Experimental runs were performed using four flat sheet commercial membranes, in the range of NF and tight UF, namely NF90, NF270, XT, and ST. They have MWCOs of 200, 400, 1000, and 10,000 Da, respectively. NF membranes, NF90 and NF270, are made of polyamide and manufactured by DOW FilmTec (Edina, MN, USA), while UF membranes, XT and ST, are made of polyethersulfone and manufactured by Synder Filtration (Vacaville, CA, USA). The main characteristics of the selected membranes are depicted in Table 1.

### 2.2. Experimental Procedure

The experiments were carried out in a plate and frame filtration unit (model Lab Unit M20) manufactured by Alfa Laval (Nakskov, Denmark), which has been described in previous works [36], using a membrane surface area of 360 cm^2^. 

Firstly, the membranes were carefully washed with a NaOH solution at pH 9.0 and 30 °C. Secondly, they were compacted by circulating distilled/deionized water (conductivity less than 2 µS cm^−1^), pressurized at 8 bar for 120 min. Then, the membranes were characterized according to their pure water permeability (*L*_PW_), as described elsewhere [37], at operating pressures (Δ*P*) of 2–8 bar, at 25 °C.

**Figure 1:**
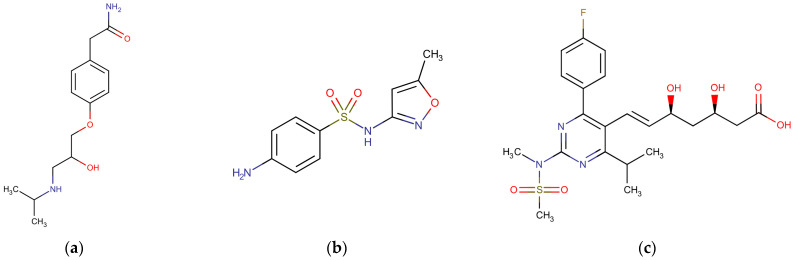
Structural formula of the PhACs studied: (**a**) atenolol; (**b**) sulfamethoxazole; and (**c**) rosuvastatin. Images from DrugBank [43,44,45].

Aqueous feed solutions with 5 mg L^−1^ of PhACs, atenolol (ATN), sulfamethoxazole (SMX), or rosuvastatin (ROS), similarly to concentrations noticed in wastewater from the pharmaceutical industry [38], have been prepared in distilled/deionized water and the pH then neutralized to 7.0 ± 0.2 with 0.1 M NaOH or HCl solutions. These PhACs were selected for the present study because, in addition to having a high consumption and being among the most detected PhACs in wastewater, they cover a molecular weight range of most PhACs and have different electrical charges (Table 2), which makes it possible to assess the electrostatic interactions with the membranes. ROS is used to control blood cholesterol levels, is among the best-selling drugs in the world, and was the third most prescribed drug in the United States in 2015 [39]. ATN is used to treat cardiovascular diseases and is also among the most prescribed drugs [40]. SMX is widely consumed and is among the most commonly found antibiotics in water and wastewater [41,42]. The physicochemical characteristics and the structural formula of the studied PhACs are presented in Table 2 and Figure 1, respectively. All the PhACs were purchased from a compounding pharmacy and had a purity level greater than 99%.

The permeation experiments were performed with 6 L of feed solution, in full recirculation mode, so that both permeate and retentate streams were continuously returned to the feed tank. Thus, the behavior of PhACs’ rejection and permeate fluxes was assessed under different operating pressures (2–8 bar), at 480 L h^−1^ (feed flow rate). A heat exchanger coupled to an ultra-thermostatic bath was used to maintain the temperature constant (25 ± 0.5 °C) in all permeation runs. Based on earlier studies [7], 30 min was used as the stabilization time in each experiment, after which feed and permeate samples were collected and analyzed. 

The permeate mass flux (*J*), in kg h^−1^ m^−2^, was calculated through Equation (1), so that *M* is the permeate mass (kg), *A* is the membrane surface area (m^2^), and *t* (h) is the permeate collection time. The apparent rejection of PhACs (*R*) was determined according to Equation (2), whose *C*_F_ and *C*_P_ are the concentrations of PhACs in the feed and in the permeate, respectively, while the intrinsic rejection (*R*′) can be calculated using Equation (3), in which *C*_M_ is the concentration of solute at the membrane surface.

(1) $$J=\frac{M}{A\;t}$$

(2) $$R\;(\%)=\frac{C_{F-}C_{P}}{C_{F}}\times 100$$

(3) $$R\prime \;(\%)=\frac{C_{M-}C_{P}}{C_{M}}\times 100$$

### 2.3. Analytical Methods

The ATN, SMX, and ROS concentrations were determined by spectrophotometric methods, whose calibration curves were obtained by measuring the absorbance at the wavelength of maximum absorbance of each compound, i.e., at 226 nm, 265 nm, and 242 nm, respectively, in a spectrophotometer T80+ UV-Vis (PG Instruments, Lutterworth, UK). The resulting calibration curves are ATN (mg L^−1^) = 28.95 × absorbance (R^2^ = 0.9997), SMX (mg L^−1^) = 14.96 × absorbance (R^2^ = 0.9998) and ROS (mg L^−1^) = 25.886 × absorbance (R^2^ = 0.9998) and are displayed in the Supplementary Materials (Figures S1–S3). All experiments and analysis were conducted in at least two replicates. The membranes were washed between each experiment by circulating distilled/deionized water or a pH 10 solution in cycles of 30 min, at 30 °C, flow rate of 480 L h^−1^, and pressures below 2 bar. The cleaning procedure was carried out until the pure water flux was restored to at least 90% of the initial value.

## 3. Theory

### 3.1. Evaluation of Concentration Polarization by Film Theory

The film theory, thoroughly described in previous works [47,48], was used to assess the concentration polarization. From the relationship between apparent (Equation (2)) and intrinsic (Equation (3)) rejections, Equation (4) is obtained, which is a linear equation *y = ax + b,* where *a* = 1/*k,* and *b* = ln((1 − *R*’)/*R*’). Therefore, from the graphical representation of ln((1 − *R*)/*R*) versus *v*_P_, a straight line, whose slope is 1/*k* and the ordinate at the origin is ln((1 − *R*’)/*R*’), is obtained. The permeation velocity (*v*_P_) is calculated by multiplying the mass permeate flux (*J*) in kg h^−1^ m^−2^ by the ratio 1/(3600*ρ*), where *ρ* is the specific mass of water in kg m^−3^. From these data, the mass transfer coefficient (*k*) at the boundary layer adjacent to the membrane and *R*’ are determined. Then, Equation (5) is used to calculate *C*_M_.

(4) $$\ln\left(\frac{1-R}{R}\right)=\ln\left(\frac{1-R^{\prime }}{R^{\prime }}\right)+\left(\frac{1}{k}\right)v_{P}$$

(5) $$C_{M}=C_{P}+\left(C_{F}-C_{P}\right)e^{\left(\frac{v_{P}}{k}\right)}$$

### 3.2. Membrane Transport Models

In the bibliography, some models have been shown to be adequate to explain the transport phenomena in membrane separation processes. In the present study, the SD and SKK models are used to evaluate the mass transfer in UF and NF membranes for aqueous solutions of PhACs, simulating the concentrations usually found in wastewaters from the pharmaceutical industry.

#### 3.2.1. Solution-Diffusion Model

The solution-diffusion model (SD) assumes that a chemical species from the feed stream dissolves in the membrane selective layer and is transported across it by diffusion, with desorption occurring at the membrane/permeate interface. This model considers that the pressure inside the membrane is uniform, so the chemical potential gradient of the chemical species permeating the membrane is represented just as a concentration gradient [49]. In fact, the SD model is already thoroughly detailed in the literature [50] and has been applied by other authors to different PhACs in forward osmosis (FO), RO, and NF membranes [51,52,53]. So, the equations used in this study to predict the behavior of membranes in removing PhACs are summarized below.

By using the experimental results of *v*_P_ in m s^−1^ and *R*’ obtained through the film theory (Section 3.1), a parameter *B*, usually called the solute permeability constant, is determined:

(6) $$B=\frac{v_{P}\left(1-R^{\prime }\right)}{R^{\prime }}.$$

*B* is characteristic of a given membrane/solute system and depends on the solute diffusion coefficient in the membrane (*D*_M_), the thickness of the membrane selective layer (*ℓ*), and the partition coefficient (Φ) so that *B* = *D*_M_Φ/*ℓ.* Therefore, the solute flux (*J*_S_) can be defined as

(7) $$J_{S}=\frac{D_{M}\Phi }{l}\left(C_{M}-C_{P}\right)=B\left(C_{M}-C_{P}\right).$$

In steady state, the solute flux across the membrane can also be represented as

(8) $$J_{S}=v_{P}C_{P}\;$$

Then, using the experimental values of *v*_P_ and *J*_S_ calculated by Equation (7), the solute concentrations in the permeate are determined by Equation (8) after a rearrangement. Later, the rejections predicted by the SD model (*R*_SD_) are calculated through Equation (2).

#### 3.2.2. Spiegler–Kedem–Katchalsky Model

The Spiegler–Kedem–Katchalsky (SKK) model explains the transport of chemical species across a membrane through a combination of convective and diffusive fluxes. It was originally designed for RO, but studies have demonstrated its potential usefulness to NF under certain conditions [54]. As the SKK model has already been extensively discussed in previous literature [55,56], we will provide a brief overview of the equations used in this study.

This model considers a relationship between the fluxes of solute (*J*_S_) and solvent—here named permeation velocity (*v*_P_)—through the membrane, as represented in Equation (9). Thus, the use of the SKK model is related to the determination of two parameters [57,58]:

- Reflection coefficient (*σ*), which is responsible for indicating the selectivity of a membrane to a given solute. *σ* = 1 represents that the membrane is impermeable to the solute (total reflection), while *σ* = 0 indicates a complete permeation of the solute.
- Solute permeability coefficient (*P*_S_), which represents the velocity of solute permeation through the membrane and is characteristic of a given membrane/solute system.

(9) $$J_{S}=P_{S}\left(C_{M}-C_{P}\right)+\left(1-\sigma \right)v_{P}C_{S}$$

By dividing Equation (9) by (*C*_M_ − *C*_P_), Equation (10) is obtained. It is a linear equation, represented as *y* = (1 − *σ*)*x* + *P*_S_, whose *y = J*_S_/(*C*_M_ − *C*_P_) and *x* = *v*_P_*C*_S_/(*C*_M_ − *C*_P_). *C*_S_ is the solute concentration inside the membrane and was calculated as the arithmetic mean between *C*_M_ and *C*_P_. Therefore, from a graphical representation of *y* versus *x,* one obtains a straight line whose slope is (1 − *σ*), and the ordinate at the origin is *P*_S_.

(10) $$\frac{J_{S}}{\left(C_{M}-C_{P}\right)}=P_{S}+\left(1-\sigma \right)\frac{v_{P}C_{S}}{\left(C_{M}-C_{P}\right)}$$

Then, from *σ* and *P*_S_ determined by the graphical method and *v*_P_ measured experimentally, the parameter *F* can be determined using Equation (11). The value of this dimensionless parameter relies on both the solute and solvent fluxes, as well as the reflection coefficient.

(11) $$F=e^{\left(1-\frac{1-\sigma }{P_{S}}\;\cdot v_{P}\right)}$$

Subsequently, the rejections predicted by the SKK model (*R*_SKK_) are calculated using Equation (12).

(12) $$R_{SKK}(\%)=\frac{\left(1-F\right)}{\left(1-\sigma F\right)}\cdot 100$$

Please note that in order to use Equations (4)–(11) from Section 3, certain unit conversions were required. As a result, in these Equations (4)–(11), rejections were expressed in decimal form, PhAC concentrations in mol m^−3^, solute flux in mol m^−2^ s^−1^, and solvent flux (permeation velocity) in m s^−1^.

## 4. Results and Discussion

**Figure 2:**
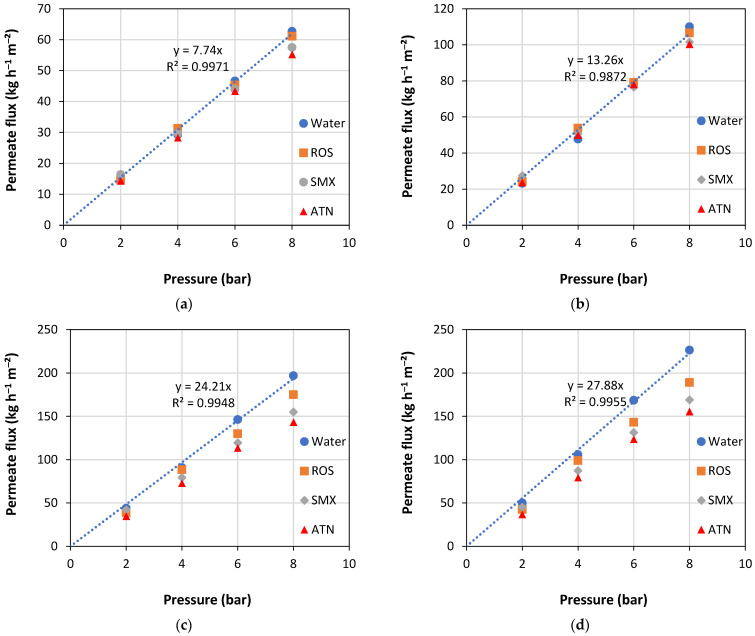
Permeate flux (*J*) as a function of operating pressure during treatment of solutions containing the PhACs for the membranes: (**a**) NF90; (**b**) NF270; (**c**) XT; and (**d**) ST.

Figure 2 displays the behavior of permeate fluxes as a function of the operating pressure, for pure water and three PhAC (ATN, SMX, and ROS) solutions, for NF90, NF270, XT, and ST membranes. As expected, pure water permeability (*L*_PW_) was dependent on MWCO and pore radius, such that membranes with higher MWCO and pore radius displayed higher *L*_PW_. Thus, the NF90, NF270, XT, and ST membranes yielded *L*_PW_ of 7.74, 13.26, 24.21, and 27.88 kg h^−1^ m^−2^ bar^−1^, respectively, these values being consistent with the ones reported in previous works [37].

The permeate fluxes for all four membranes and all three PhAC solutions investigated vary linearly with operating pressure, and the corresponding straight-line slopes for the NF membranes are practically the same as the pure water. For UF membranes, they are below that the ones with pure water (Figure 2). Furthermore, as membrane MWCO and pressure rise, the deviation between permeate fluxes with pure water and with solutions of PhACs also increases, due to the increase in concentration polarization phenomenon, especially for the UF membranes. The permeate flux was also dependent on the PhAC present in the feed solution. Thus, the solution containing the positively charged compound (ATN) showed the lowest permeate fluxes, followed by the solution containing the negatively charged and lower MW compound (SMX), while the one containing the negatively charged and higher MW compound (ROS) showed higher permeate fluxes and closer to the values achieved with pure water. Similar performance, with a reduction in the permeate flux during the treatment of solutions containing PhAC of opposite charge to the membrane, was observed in previous works, however, with another PhAC, norfloxacin [5].

**Figure 3:**
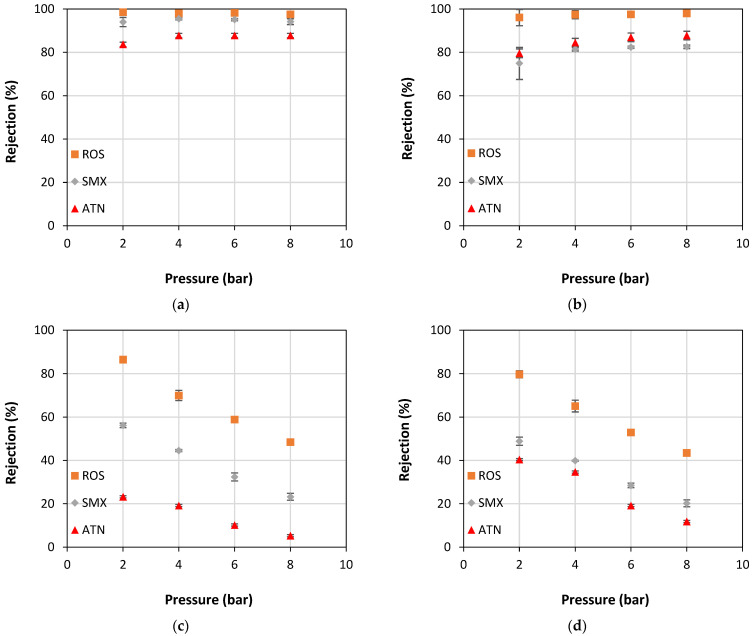
Apparent rejection (*R*) of ROS, SMX, and ATN PhACs as a function of operating pressure for membranes: (**a**) NF90; (**b**) NF270; (**c**) XT; and (**d**) ST.

The behavior of apparent rejection to the PhACs ROS, SMX, and ATN as a function of operating pressure for the NF90, NF270, XT, and ST membranes is illustrated in Figure 3a–d, respectively. As a rule, a dependence of the rejection can be observed with the type of PhAC, membrane MWCO, and operating pressure assessed. It is important to point out that the UF membranes (Figure 3c,d) showed a greater variation in rejections considering all parameters investigated. Indeed, one can note two completely distinct behaviors. NF membranes demonstrate high apparent rejections of all PhACs regardless of the operating pressure, while UF membranes show a significant decrease in the apparent rejection as the pressure increases.

The tightest membrane, NF90, exhibited the highest apparent rejections for the three PhACs evaluated and the lowest variability in rejections under the conditions tested, reaching values between 84% and 98%. So, for this membrane, the rejection was independent of pressure, but dependent on the PhAC evaluated, reaching mean values of 98.02 ± 0.43%, 94.71 ± 1.13%, and 86.73 ± 2.04%, for ROS, SMX, and ATN, respectively (Figure 3a). As shown in Table 1, the NF90 membrane has a 200 Da MWCO and a negative surface charge [5], while ROS, SMX, and ATN have MW of 482 Da, 253 Da, and 266 Da, and charges −1, −1, and +1, respectively, at the pH studied (pH 7). As the NF90 membrane has a MWCO lower than the MW of the PhACs studied, total rejection of these compounds could be expected, which was not observed. It can be seen that both the MW of the PhAC and its charge had an influence on rejection, the latter being the most important in this particular case. For the two negatively charged PhACs, the rejection values were very high, above 95%. On the other hand, even the ATN (266 Da) having an MW slightly higher than the SMX, but with a positive charge, showed rejections about 8% lower than the SMX, which has a negative charge, thus demonstrating an important role of the electrostatic interactions in the rejection of PhACs by tight nanofiltration membranes such as NF90. These results are consistent with those seen in previous studies performed with this NF membrane, where the positively charged PhAC had lower rejections than the negatively charged, despite both having a very similar MW [24].

The NF270 membrane, in turn, despite having been considered a loose NF membrane [33], with MWCO of 400 Da, also achieved high apparent rejections to the studied PhACs but slightly lower than those reached with the NF90 membrane. For ROS (482 Da), which presents MW higher than the MWCO of the NF270 membrane, the rejection values remained constant in the pressure range studied, around 97.22 ± 1.72%. On the other hand, for ATN and SMX, which have MW lower than the MWCO of the NF270 membrane, rejections were lower than those obtained for ROS, and it was dependent on the operating pressure. For these two PhACs, a slight increase in rejection is noticed as the operating pressure increases, going from about 75% and 79% rejection at 2 bar to about 83% and 88% rejection at 8 bar, respectively (Figure 3b). 

As can be seen in Figure 3c,d, the UF membranes, XT and ST, showed very significant apparent rejection variation to PhACs as a function of both the PhAC and the applied pressure. These membranes are made of polyethersulfone and have MWCO of 1000 Da and 10,000 Da, respectively, therefore with MWCO greater than the MW of the PhACs (ROS: 482 Da, SMX: 253 Da, ATN: 266 Da), thus justifying their lower rejections to these compounds. Moreover, given the high difference between the MWCO of the ST membrane (10,000 Da) and the MW of the PhACs, even lower rejections would be expected.

**Figure 4:**
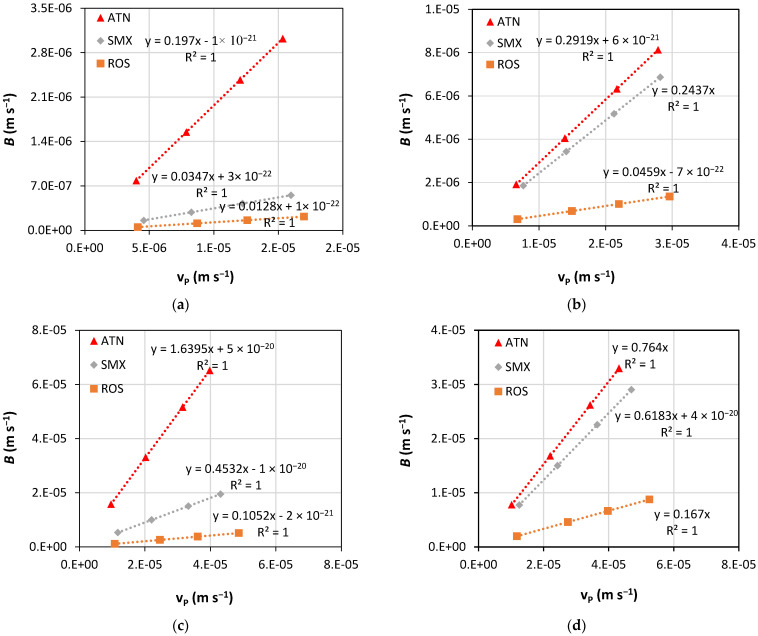
Variation of the solute permeability coefficient (*B*) for ATN, SMX, and ROS as a function of the permeation velocity (*v*_P_) for the membranes: (**a**) NF90, (**b**) NF270, (**c**) XT, and (**d**) ST.

The relatively high ROS rejection rates displayed by the XT (48–86%) and ST (43–80%) membranes are related to electrostatic repulsion forces between the ROS—which has a negative charge (−1) at neutral pH (pH of the solution studied)—and the aforementioned membranes, which also have negative surface charges at neutral pH [59]. The same behavior was also observed with SMX, which also has a negative charge (−1) at neutral pH; however, as this solute has a MW corresponding to 55% of the ROS’s MW, the rejections were lower, ranging from 23% to 56% and from 23% to 49% for XT and ST membranes, respectively. Furthermore, these UF membranes presented a reduction in ROS and SMX rejection rates with rising operating pressure. This could be because the higher the operating pressure is, the lower the influence of electrostatic repulsion interactions and, therefore, lower rejection values. In addition, there is an increase in concentration polarization (see Table S1) that leads to higher concentrations on the membrane surface and lower rejections overall (see Figure 4c,d). On the other hand, studies have reported that when the solute flux is directly dependent on pressure, its permeation is controlled by convection, whereas for pressure-independent solute fluxes, permeation is governed by diffusion [60].

When filtering the solution containing ATN, the lowest rejections by the XT and ST membranes among the three studied PhACs were observed, as well as a drop in rejection with increasing operating pressure. This behavior may be associated with the fact that ATN is positively charged (+1) at the pH studied, causing an attraction between it and the membranes (which are negatively charged), thus increasing the solute/membrane affinity and its subsequent permeation through the membrane. In light of these results, one may speculate that the removal of ATN decreases as the pressure increases because the force of the applied pressure overcomes the adsorption forces that kept the ATN adsorbed to the membranes, and, thus, it crosses the UF membranes towards the permeate stream.

As the results displayed in Figure 2 and Figure 3 indicate the occurrence of concentration polarization, especially for UF membranes, this phenomenon was evaluated in the present work by the pressure variation method, which was thoroughly described in previous works [24]. Therefore, Equations (4) and (5) were used to quantify the concentration polarization, so that the first allowed the determination of *k* and *R*’, while the second was used to calculate *C*_M_. The *k* and *R*’ values are presented in Table 3, *C*_M_ is depicted in Table S1, and the graphical representation of Equation (4) is illustrated in Figure S4.

In Table S1, an increase in concentration polarization is observed with increasing pressure for all PhACs and all membranes. A greater concentration polarization is also observed in membranes with higher hydraulic permeability, that is, in UF membranes. In the most striking case, at the pressure of 8 bar, the ST membrane showed a concentration of ROS on the surface of the membrane (*C*_M_) 4.19 times greater than that found in the bulk feed solution (*C*_F_). Under these conditions, a value as high as 10.22 is achieved for the concentration polarization module, whose definition is (*C*_M_ − *C*_P_)/(*C*_F_ − *C*_P_). For NF membranes, on the other hand, the concentration polarization module was between 0.09 and 0.85, indicating a low incidence of this undesired phenomenon (see Table S1). It is important to note that these results are consistent with the ones shown in Figure 2, where one can see greater deviations between the permeate fluxes obtained with solutions containing PhACs and those achieved with pure water for UF membranes compared to NF membranes. Previous studies also demonstrated that membranes with higher permeate fluxes were subject to more intense concentration polarization phenomena [48]. Moreover, the mass transfer model based on concentration polarization showed a good fit for the UF membranes, obtaining mean *k* values of 2.16 (±0.31) × 10^−5^ m s^−1^ (Table 3). The very small slopes obtained for the NF membranes (see Figure S4a–b), especially for the tightest membrane (NF90), resulted in higher *k* values and worse fits (R^2^ values) when compared to the values obtained with the UF membranes. 

Figure 4 illustrates the behavior of the solute permeability coefficient (*B*) for ATN, SMX, and ROS as a function of permeation velocity (*v*_P_) for the four membranes assessed. One can observe that the solute permeability coefficient increased linearly with the permeation velocity for all three PhACs tested. It is also observed that higher *B* values are associated with greater deviation in permeate fluxes with PhAC solutions compared to pure water fluxes (Figure 2). Higher *B* values are also related to lower apparent rejection rates (Figure 3), possibly due to the enhancement of concentration polarization. This phenomenon is also observed in other studies [48,61], where high permeate fluxes can lead to an increase in concentration polarization and, consequently, to a greater interaction between solutes and the membrane. The behavior of the solute permeability coefficient is also related to electrostatic solute/membrane interactions. All membranes have a negative charge, and the experiments with a positively charged solute (ATN) resulted in the highest values of *B*, being in agreement with the results found in previous studies [24]. In the most notable case, at the pressure of 8 bar, the XT membrane presented the value of *B* for ATN (charge +1) about 12 times greater than that found for ROS, which is negatively charged.

**Figure 5:**
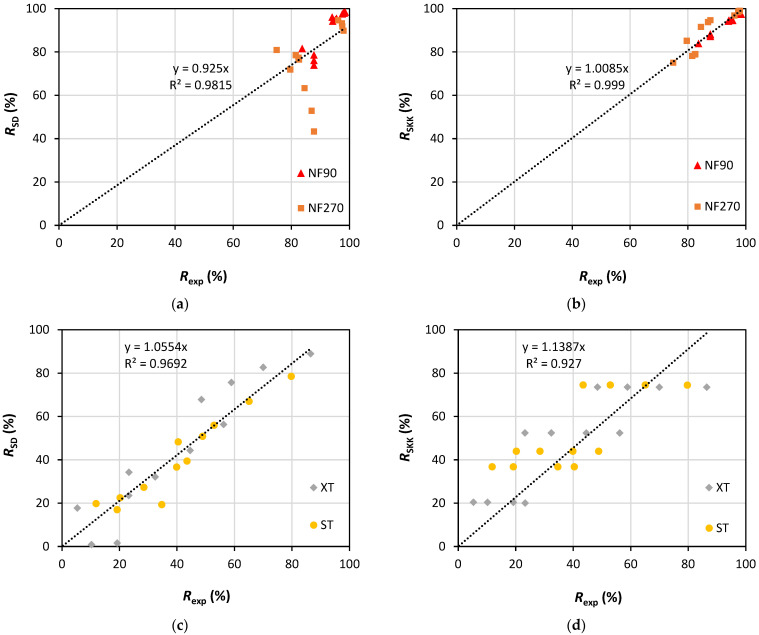
Relation between experimental rejection (*R*_exp_) and rejections calculated by solution diffusion (*R*_SD_) and Spiegler–Kedem–Katchalsky (*R*_SKK_) models for NF (**a**,**b**) and UF (**c**,**d**) membranes.

Figure 5 shows the values of the experimental apparent rejections as a function of the rejections predicted by the solution/diffusion and the SKK models for the three PhACs and the four membranes studied. For NF membranes, a very good fit (R^2^ = 0.999) of the experimental results with the values predicted by the SKK model and a worse fit with the SD model is observed. This behavior may be associated with the fact that the SD model considers the solute flux as being diffusive, while the SKK model considers the transport of solute through the membranes by diffusion/convection, which influences the solute rejection (Figure 3a,b). Other studies report greater solute rejection at higher operating pressures in agreement with the SKK model [62]. On the other hand, for UF membranes, a good fit (R^2^ 0.9692) of the experimental rejections with the rejections predicted by the SD model and a poor fit with the SKK model was observed. Indeed, these results are surprising since UF membranes are traditionally known to exhibit predominantly convective transport. One can speculate that the good fit of the UF membrane results with the SD model may be associated with the incidence of intense concentration polarization, whose occurrence has already been discussed and is illustrated in Figure 2 and Table S1. Thus, a high solute concentration in the boundary layer adjacent to the membrane surface (*C*_M_) could increase the solute diffusion through the membrane towards the permeate stream.

## 5. Conclusions

The present work investigated ultrafiltration and nanofiltration under different operating conditions for the removal of three different PhACs from water. The ultrafiltration membranes (XT and ST) displayed the highest permeate fluxes with lower rejection rates for the studied PhACs, 5–86%. The rejection rates were dependent upon:

(i) Operating pressure: the higher the operating pressure is, the lower the rejection rate;
(ii) Surface charge of the PhAC: solutes with a negative charge, the same as of the membranes, presented higher rejections than those with a positive charge, despite having similar MW;
(iii) MW of PhAC: among the solutes with a negative charge, the one with the highest MW had the highest rejections.

It is important to highlight that electrostatic interactions were a crucial phenomenon also in ultrafiltration, while a membrane with MWCO about 20 times greater than the MW of the solute achieved rejections to this solute of up to 80%.

Both nanofiltration membranes evaluated, NF90 and NF270, showed high rejections for all PhACs studied, 75–98%, being the lowest rejections for ATN, which has a positive charge. Considering the parameters of productivity and selectivity, the NF270 membrane was the most efficient, as it presented permeate fluxes as high as 100 kg h^−1^ m^−2^ and rejections higher than 80% for all three PhACs at 8 bar of transmembrane pressure. In addition, under the conditions evaluated, no fouling was observed, which provides a longer lifetime for the membranes. And, a concentration polarization module below 0.85 for NF and up to 10.22 for UF was achieved. Therefore, in the present study, nanofiltration was the most recommended membrane technology, but tight ultrafiltration also showed significant removal of PhACs from water when low operating pressures were applied.

## Figures and Tables

**Table 1:** Characteristics of the selected membranes.

Parameter	Membrane Type			
	ST	XT	NF270	NF90
Membrane active layer	Polyethersulfone ^a^	Polyethersulfone ^a^	Semi-aromatic Polyamide ^a,d^	Fully aromatic Polyamide ^a,d^
MWCO (Da)	10,000 ^a^	1000 ^a^	400 ^f^	200 ^f^
pH operating range	3–9 ^a^	3–9 ^a^	3–10 ^a^	2–11 ^a^
Maximum operating pressure (bar)	8.3 ^a^	8.3 ^a^	41 ^a^	41 ^a^
Zeta potential at pH 7 (mV)	n.a.	−20 ^e^	−22 ^c^; −29 ^d^	−25 ^c^; −32 ^d^
Contact angle (°)	72.2 ^b^	63.5 ^b^	54.3 ^d^	61.6 ^d^
Pore radius (nm)	7.84 ^b^	5.88 ^b^	0.44 ^d^	0.36 ^d^
Manufacturer	Synder Filtration	Synder Filtration	Dow—FilmTec	Dow—FilmTec

n.a.: not available; ^a^ from manufacturer; ^b^ [32]; ^c^ [33]; ^d^ [34]; ^e^ [35]; ^f^ [24].

**Table 2:** Physicochemical characteristics of atenolol (ATN), sulfamethoxazole (SMX), and rosuvastatin (ROS).

Physicochemical Characteristic	ATN	SMX	ROS
Molecular formula	C_14_H_22_N_2_O_3_ ^a^	C_10_H_11_N_3_O_3_S ^b^	C_22_H_28_FN_3_O_6_S ^c^
Molecular weight (Da)	266 ^a^	253 ^b^	482 ^c^
pKa	9.6 ^a^	1.97; 6.16 ^b^	4.0 ^c^
log K_OW_	0.16 ^a^	0.89 ^b^	0.13 ^c^
*D* (×10^−10^ m^2^ s^−1^, 25 °C)	5.25 ^d^	6.17 ^d^	4.91 ^f^
Stokes radius (nm)	0.46 ^d^	0.40 ^d^	0.50 ^e^
Surface charge at pH 7	+1 ^a^	−1 ^b^	−1 ^c^

^a^ [43]; ^b^ [44]; ^c^ [45]; ^d^ [34]; ^e^ [46]; ^f^ Calculated by Wilke and Chang equation.

**Table 3:** Intrinsic rejections (*R*’) and mass transfer coefficients (*k*) obtained for the permeation of the PhACs solutions through NF90, NF270, XT, and ST membranes.

Membrane	ATN		SMX		ROS	
	*k* × 10^−5^ (m s^−1^)	*R*′ (%)	*k* × 10^−5^ (m s^−1^)	*R*′ (%)	*k* × 10^−5^ (m s^−1^)	*R*′ (%)
NF90	3.75	88	2.87	97	2.66	99
NF270	3.51	77	18.4	80	3.65	96
XT	1.77	38	2.17	69	2.05	90
ST	1.98	57	2.57	62	2.48	86

## Data Availability

The data that support the findings of this study are available from the corresponding author upon reasonable request.

## Supplementary Materials

The following supporting information can be downloaded at: https://www.mdpi.com/article/10.3390/membranes13080743/s1, Figure S1. Calibration curve for determination of atenolol (ATN) by spectrophotometric method. ATN concentration versus absorbance at 226 nm (wavelength of maximum absorption); Figure S2. Calibration curve for determination of sulfamethoxazole (SMX) by spectrophotometric method. SMX concentration versus absorbance at 265 nm (wavelength of maximum absorption); Figure S3. Calibration curve for determination of rosuvastatin (ROS) by spectrophotometric method. ROS concentration versus absorbance at 242 nm (wavelength of maximum absorption); Figure S4. Variation of ln((1 − *R*)/*R*) with the permeation velocity (*v*_P_) of PhACs solutions for membranes: (a) NF90; (b) NF270; (c) XT; and (d) ST; Figure S5. Variation of *J*_S_/(*C*_F_ − *C*_P_) with (*v*_P_*C*_S_)/(*C*_F_ − *C*_P_) of PhACs solutions for membranes: (a) NF90; (b) NF270; (c) XT; and (d) ST. SKK model; Table S1. Mean concentration of PhACs (ATN, SMX, and ROS) in the bulk feed solution (*C*_F_), in the permeate (*C*_P_), at the boundary layer adjacent to the membrane surface (*C*_M_) determined by film theory, as well as the concentration polarization module assessed in the different operating pressures; Table S2. Values of experimental (apparent), intrinsic, and calculated rejections by SD and SKK models for ATN, SMX, and ROS at pressures from 2 to 8 bar.
