# Supplementary material for: Ultrafiltration and Nanofiltration for the Removal of Pharmaceutically Active Compounds from Water: The Effect of Operating Pressure on Electrostatic Solute—Membrane Interactions
Source: Membranes (Basel). 2023 Aug 19;13(8):743. doi: 10.3390/membranes13080743 (PMC10456375; doi:10.3390/membranes13080743)
Supplement: Supplementary file 1 [file membranes-13-00743-s001.zip › membranes-2484235-supplementary.pdf]

**Supplementary Material**

# Ultrafiltration and Nanofiltration for the Removal of Pharmaceutically Active Compounds from Water: The Effect of Operating Pressure on Electrostatic Solute-Membrane Interactions

Alexandre Giacobbo[1,2,]*, Isabella Franco Pasqualotto[1], Rafael Cabeleira de Coronel Machado Filho[1], Miguel Minhalma[2,3], Andréa Moura Bernardes[1], Maria Norberta de Pinho[2,4]*

[1] *Post-Graduation Program in Mining, Metallurgical and Materials Engineering (PPGE3M), Federal University of Rio Grande do Sul (UFRGS), Av. Bento Gonçalves n. 9500, CEP: 91.509-900 Porto Alegre, Brazil. E-mail: alexandre_giacobbo@yahoo.com.br*

[2] *Centre of Physics and Engineering of Advanced Materials (CeFEMA), Instituto Superior Técnico, University of Lisbon, Av. Rovisco Pais, n. 1, 1049-001 Lisbon, Portugal*

[3] *Chemical Engineering Department, Instituto Superior de Engenharia de Lisboa, Instituto Politécnico de Lisboa, Rua Conselheiro Emídio Navarro, 1, 1959-007 Lisboa, Portugal*

[4] *Chemical Engineering Department, Instituto Superior Técnico, University of Lisbon, Av. Rovisco Pais, n. 1, 1049-001 Lisbon, Portugal*

[*] *Author to whom correspondence should be addressed*

**Supplementary Material includes 5 figures and 2 tables.**

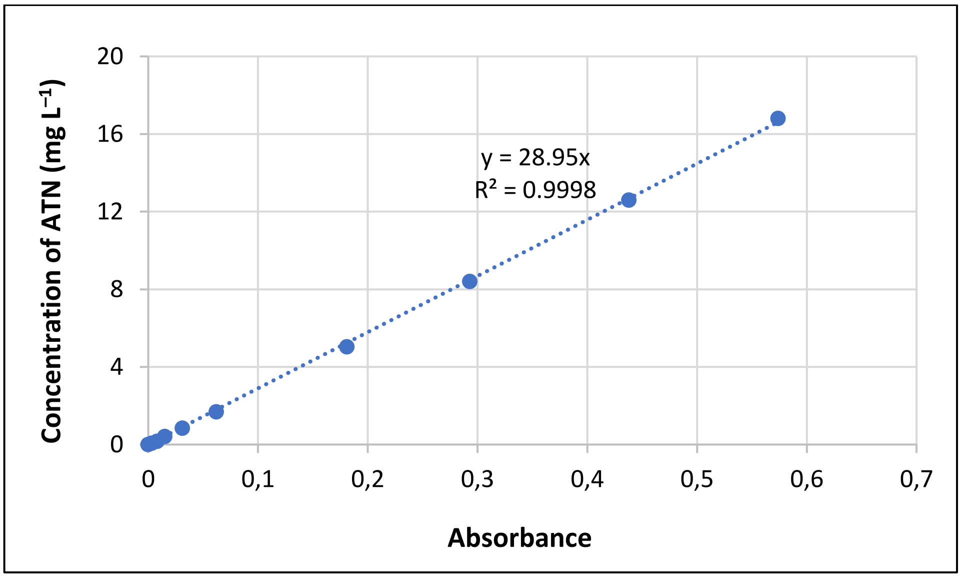


**Figure S1.** Calibration curve for determination of atenolol (ATN) by spectrophotometric method. ATN concentration versus absorbance at 226 nm (wavelength of maximum absorption).

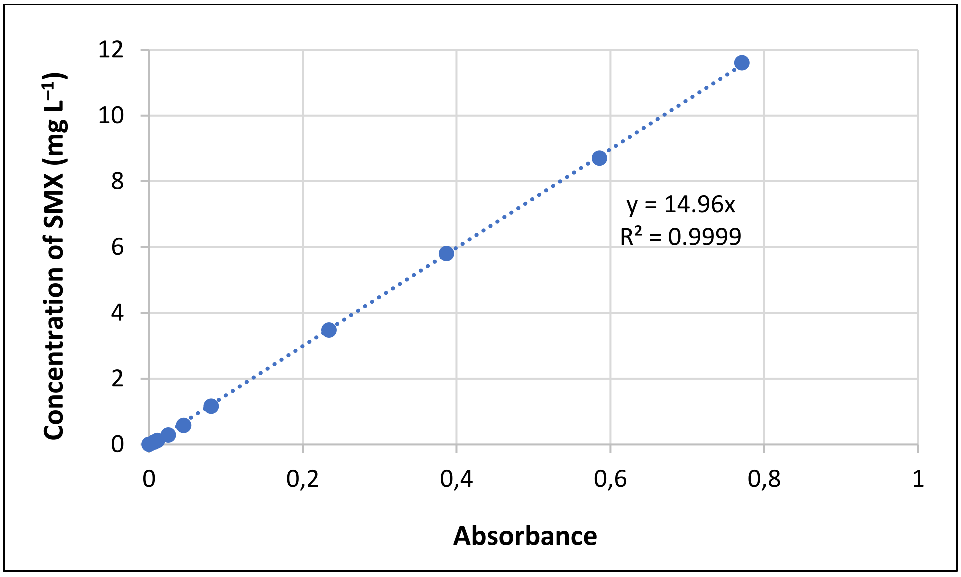


**Figure S2.** Calibration curve for determination of sulfamethoxazole (SMX) by spectrophotometric method. SMX concentration versus absorbance at 265 nm (wavelength of maximum absorption).

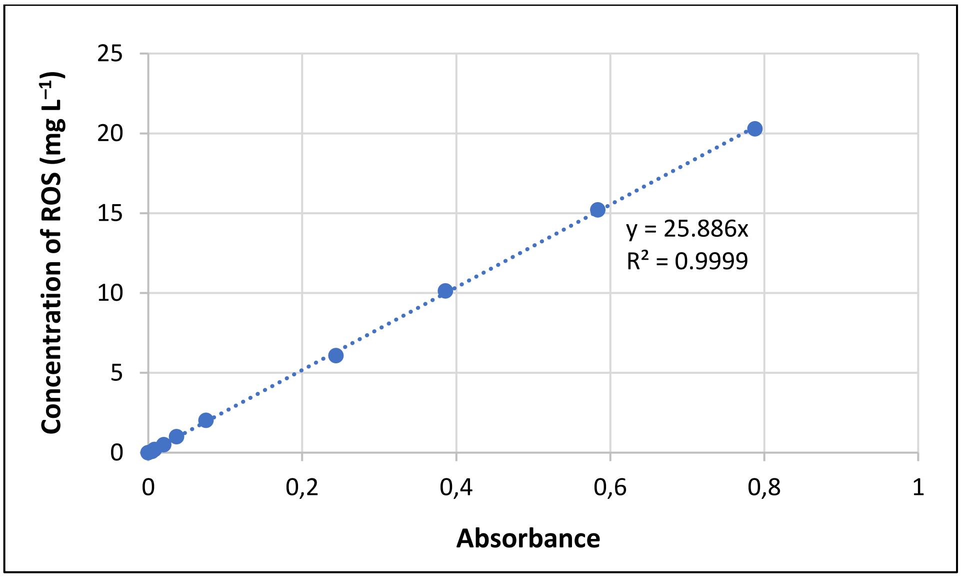


**Figure S3.** Calibration curve for determination of rosuvastatin (ROS) by spectrophotometric method. ROS concentration versus absorbance at 242 nm (wavelength of maximum absorption).

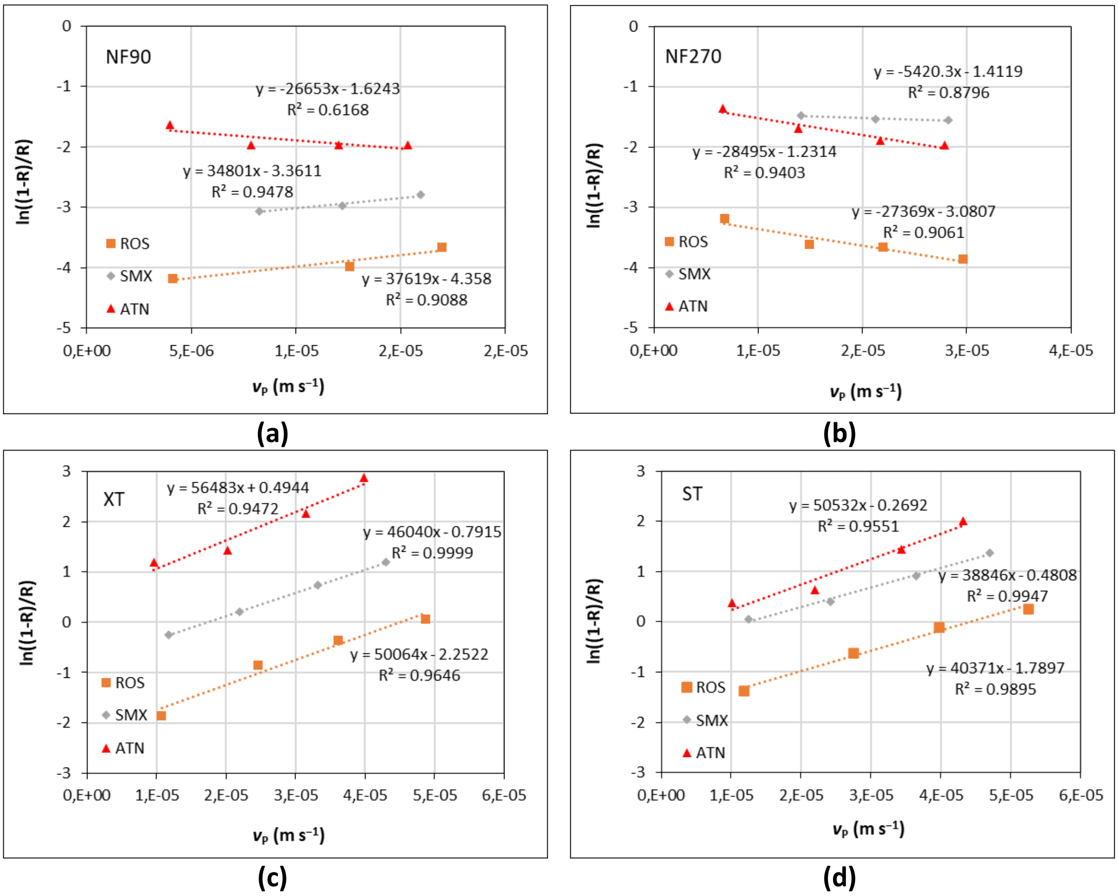


**Figure S4.** Variation of ln((1 – R)/R) with the permeation velocity ($v_P$) of PhACs solutions for membranes: a) NF90; b) NF270; c) XT; and d) ST.

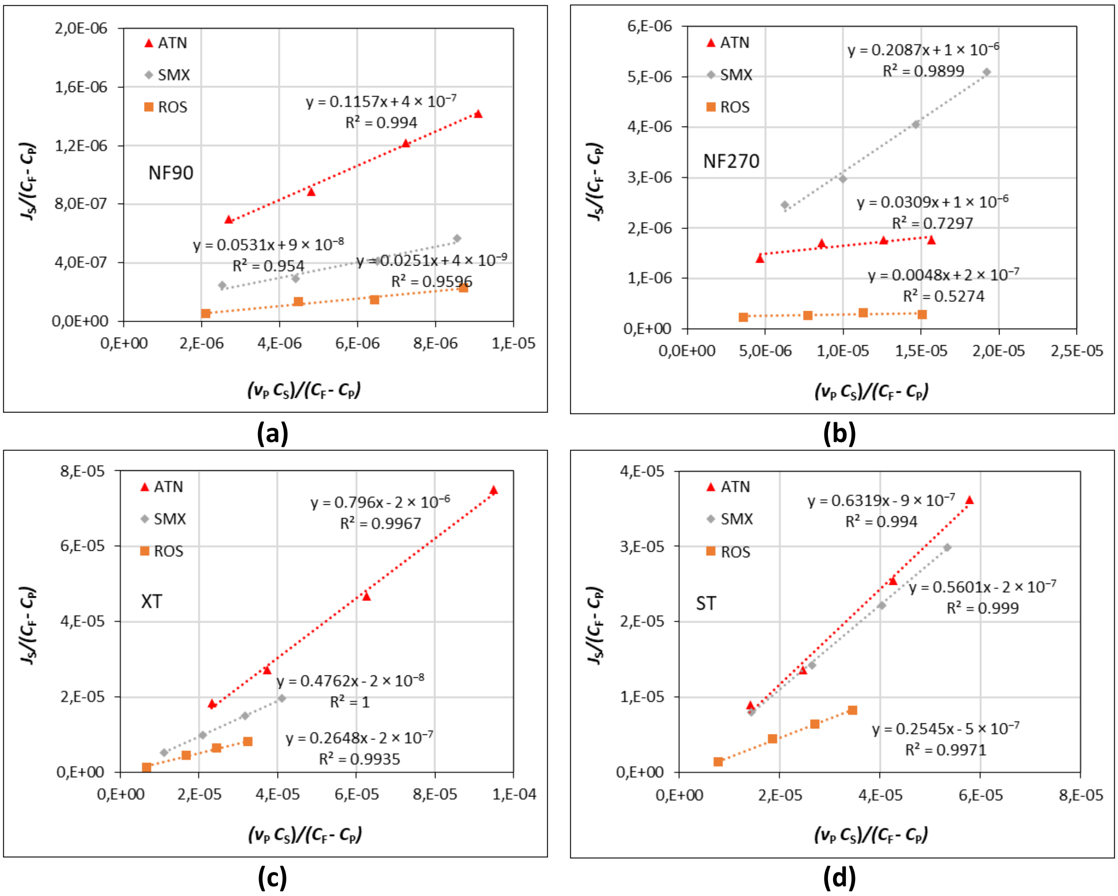


**Figure S5.** Variation of $J_S/(C_F - C_P)$ with $(v_P C_S)/(C_F - C_P)$ of PhACs solutions for membranes: a) NF90; b) NF270; c) XT; and d) ST. SKK model.

**Table S1.** Mean concentration of PhACs (ATN, SMX and ROS) in the bulk feed solution ($C_F$), in the permeate ($C_P$), at the boundary layer adjacent to the membrane surface ($C_M$) determined by Film Theory, as well as the concentration polarization module assessed in the different operating pressures.

| **PhAC** | **ΔP** | $C_F$ | **NF90** | | | **NF270** | | | **XT** | | | **ST** | | |
|---|---|---|---|---|---|---|---|---|---|---|---|---|---|---|
| | | | $C_M$ | $C_P$ | $\frac{(C_M - C_P)}{(C_F - C_P)}$ | $C_M$ | $C_P$ | $\frac{(C_M - C_P)}{(C_F - C_P)}$ | $C_M$ | $C_P$ | $\frac{(C_M - C_P)}{(C_F - C_P)}$ | $C_M$ | $C_P$ | $\frac{(C_M - C_P)}{(C_F - C_P)}$ |
| | **(bar)** | **(mg L$^{-1}$)** | **(mg L$^{-1}$)** | **(mg L$^{-1}$)** | | **(mg L$^{-1}$)** | **(mg L$^{-1}$)** | | **(mg L$^{-1}$)** | **(mg L$^{-1}$)** | | **(mg L$^{-1}$)** | **(mg L$^{-1}$)** | |
| ATN | 2 | 3.55 | 3.88 | 0.58 | 0.54 | 4.13 | 0.72 | 0.70 | 4.15 | 2.72 | 1.09 | 4.52 | 2.12 | 1.43 |
| | 4 | 3.55 | 4.28 | 0.43 | 0.47 | 5.01 | 0.55 | 0.69 | 5.00 | 2.87 | 1.72 | 6.07 | 2.32 | 2.45 |
| | 6 | 3.55 | 4.73 | 0.43 | 0.53 | 6.19 | 0.46 | 0.75 | 5.34 | 3.19 | 1.93 | 6.73 | 2.87 | 3.12 |
| | 8 | 3.55 | 5.12 | 0.43 | 0.57 | 7.33 | 0.43 | 0.84 | 5.14 | 3.36 | 1.69 | 6.86 | 3.13 | 3.29 |
| SMX | 2 | 3.94 | 4.58 | 0.24 | 0.26 | 4.06 | 0.99 | 0.77 | 5.52 | 1.73 | 1.66 | 5.15 | 2.01 | 1.60 |
| | 4 | 3.94 | 5.19 | 0.18 | 0.23 | 4.19 | 0.73 | 0.64 | 7.02 | 2.18 | 2.68 | 6.40 | 2.37 | 2.43 |
| | 6 | 3.94 | 5.93 | 0.19 | 0.28 | 4.34 | 0.69 | 0.64 | 8.56 | 2.66 | 3.99 | 7.45 | 2.82 | 3.31 |
| | 8 | 3.94 | 6.70 | 0.23 | 0.38 | 4.48 | 0.69 | 0.66 | 9.67 | 3.03 | 5.11 | 8.08 | 3.14 | 3.94 |
| ROS | 2 | 4.99 | 5.81 | 0.08 | 0.09 | 5.96 | 0.20 | 0.23 | 5.96 | 0.68 | 0.72 | 7.42 | 1.01 | 1.30 |
| | 4 | 4.99 | 6.88 | 0.10 | 0.14 | 7.45 | 0.13 | 0.19 | 9.73 | 1.50 | 2.47 | 11.60 | 1.74 | 3.44 |
| | 6 | 4.99 | 7.96 | 0.09 | 0.15 | 9.01 | 0.13 | 0.22 | 13.58 | 2.05 | 4.74 | 15.50 | 2.35 | 6.19 |
| | 8 | 4.99 | 9.34 | 0.13 | 0.23 | 11.09 | 0.10 | 0.23 | 17.84 | 2.57 | 7.87 | 20.91 | 2.82 | 10.22 |

**Table S2.** Values of experimental (apparent), intrinsic, and calculated rejections by SD and SKK models for ATN, SMX, and ROS at pressures from 2 to 8 bar.

| PhAC | ΔP (bar) | NF90 | | | | NF270 | | | | XT | | | | ST | | | |
|---|---|---|---|---|---|---|---|---|---|---|---|---|---|---|---|---|---|
| | | Exp. | Film | SD | SKK | Exp. | Film | SD | SKK | Exp. | Film | SD | SKK | Exp. | Film | SD | SKK |
| | | *R* (%) | *R'* (%) | $R_{SD}$ (%) | $R_{Skk}$ (%) | *R* (%) | *R'* (%) | $R_{SD}$ (%) | $R_{Skk}$ (%) | *R* (%) | *R'* (%) | $R_{SD}$ (%) | $R_{Skk}$ (%) | *R* (%) | *R'* (%) | $R_{SD}$ (%) | $R_{Skk}$ (%) |
| ATN | 2 | 83.67 | 83.54 | 81.66 | 83.95 | 79.59 | 77.41 | 71.97 | 85.24 | 23.27 | 37.89 | 34.28 | 20.05 | 40.41 | 56.69 | 48.32 | 36.79 |
| | 4 | 87.76 | | 78.68 | 87.27 | 84.49 | | 63.37 | 91.63 | 19.18 | | 1.60 | 20.39 | 34.69 | | 19.35 | 36.81 |
| | 6 | 87.76 | | 76.17 | 88.11 | 86.94 | | 52.92 | 93.87 | 10.20 | | 0.86 | 20.40 | 19.18 | | 17.00 | 36.81 |
| | 8 | 87.76 | | 73.98 | 88.31 | 87.76 | | 43.33 | 94.77 | 5.31 | | 17.73 | 20.40 | 11.84 | | 19.83 | 36.81 |
| SMX | 2 | 94.01 | 96.65 | 96.18 | 94.32 | 74.91 | 80.41 | 80.98 | 75.13 | 56.18 | 68.82 | 56.35 | 52.38 | 48.88 | 61.79 | 50.84 | 43.99 |
| | 4 | 95.51 | | 95.58 | 94.65 | 81.46 | | 78.57 | 78.22 | 44.57 | | 44.35 | 52.38 | 39.89 | | 36.67 | 43.99 |
| | 6 | 95.13 | | 94.95 | 94.69 | 82.40 | | 77.47 | 78.93 | 32.40 | | 32.15 | 52.38 | 28.46 | | 27.34 | 43.99 |
| | 8 | 94.19 | | 94.30 | 94.69 | 82.58 | | 76.55 | 79.08 | 23.22 | | 23.52 | 52.38 | 20.22 | | 22.49 | 43.99 |
| ROS | 2 | 98.48 | 98.74 | 98.53 | 97.49 | 96.06 | 95.61 | 96.69 | 96.88 | 86.45 | 90.48 | 88.87 | 73.52 | 79.72 | 85.69 | 78.55 | 74.50 |
| | 4 | 97.94 | | 98.26 | 97.49 | 97.38 | | 93.27 | 98.42 | 69.96 | | 82.66 | 73.52 | 65.08 | | 67.02 | 74.55 |
| | 6 | 98.15 | | 97.98 | 97.49 | 97.49 | | 91.82 | 98.84 | 58.88 | | 75.70 | 73.52 | 52.91 | | 55.99 | 74.55 |
| | 8 | 97.49 | | 97.64 | 97.49 | 97.94 | | 89.88 | 99.06 | 48.45 | | 67.83 | 73.52 | 43.47 | | 39.47 | 74.55 |
